# Supplementary material for: The Greek Jefferson Scale of Empathy—Medical Student Version (JSE-S): Psychometric Properties and Its Associated Factors
Source: Behav Sci (Basel). 2024 Feb 28;14(3):195. doi: 10.3390/bs14030195 (PMC10968493; doi:10.3390/bs14030195)
Supplement: Supplementary file 1 [file behavsci-14-00195-s001.zip › behavsci-2829682-supplementary.pdf]

The Greek version of the JSE is protected by copyright. It can be made available by the copyright holder upon reasonable request. The copyright holder is "Thomas Jefferson University (email: empathy@jefferson.edu)"
